# Maternal diet and fatty acids in human milk: results of the MELK study

**DOI:** 10.1007/s00394-026-04083-8

**Published:** 2026-08-25

**Authors:** Inga Petersohn, Peter L. Zock, Karen Knipping, Elske M. Brouwer-Brolsma, Edith J. M. Feskens

**Affiliations:** 1 Division of Human Nutrition and Health, Wageningen University and Research, Wageningen, The Netherlands; 2 Ausnutria B.V., Zwolle, The Netherlands

**Keywords:** Human milk, Fatty acid, Diet, Macronutrients, Micronutrients

## Abstract

**Purpose:**

Human milk is the preferred nutrition for infants. Maternal diet influences its fatty acid composition particularly fish-derived fatty acids and polyunsaturated fatty acids (PUFAs). Evidence for other fatty acids and associations with micronutrient intake remains inconsistent. This study examines associations between maternal macro- and micronutrient intake and human milk fatty acid composition.

**Methods:**

Data of 109 breastfeeding mothers was collected. Dietary intake was assessed using repeated 24-h food records and a short food frequency questionnaire. Nutrient intakes were calculated using the Dutch food composition database. Human milk samples were collected at 6–8 weeks postpartum following a standardized protocol, and fatty acid concentrations were analyzed using ISO16958:2015. Associations between maternal diet and human milk fatty acids were evaluated using beta regression. The MELK study is registered at the ISRCTN registry (ISRCTN35735283).

**Results:**

Higher maternal intakes of n-3 PUFAs, n-6 PUFAs, and trans fatty acids (TFAs) were associated with higher proportions in milk. Milk total saturated fatty acids (SFA) were inversely associated with most dietary fatty acid groups. At the individual level, higher SFA intake was positively associated with several milk SFAs, while higher TFA intake was linked to higher palmitic acid in milk, intake of n-3 PUFA showed the opposite pattern. Higher micronutrient intake was associated with increased milk PUFA levels and lower SFA and TFA levels.

**Conclusion:**

This study provides further detail on links between maternal dietary fatty acids and their levels in human milk, and suggests that micronutrient and vitamin intake may also influence milk fatty acids.

**Supplementary Information:**

The online version contains supplementary material available at https://doi.org/10.1007/s00394-026-04083-8.

## Introduction

Breastfeeding offers a safe, nutritionally complete, readily available, and cost-effective source of nourishment for infants. Thus, the World Health Organization and the United Nations Children’s Fund advise that mothers should whenever possible exclusively breastfeed their infants for the first six months of life, and continue breastfeeding alongside the introduction of solid foods for up to two years [1].

The fatty acid profile of human milk is linked to several aspects of infant health and development, with evidence differing by fatty acid class. Saturated fatty acids (SFAs) make up a large share of total human milk fat and contribute substantially to infant dietary energy intake [2]. Palmitic acid is the main long-chain SFA and is typically esterified at the sn-2 position of triacylglycerols; studies show that this positioning improves fat and calcium absorption compared with formulas where palmitic acid is mainly at sn-1 and sn-3 [3, 4]. In addition to long-chain SFAs, short-chain fatty acids (SCFAs) such as butyrate, formic acid, and acetate present in human milk have been inversely associated with infant adiposity, suggesting a potential protective effect against excess weight gain [5]. Monounsaturated fatty acids (MUFAs), represent a major proportion of human milk fatty acids and primarily contribute to the energy content, with oleic acid being the most abundant MUFA [6]. Although links to infant growth have been studied, no specific developmental role of MUFA is confirmed [7]. Polyunsaturated fatty acids (PUFAs) show the strongest functional associations. Docosahexaenoic acid (DHA) is linked to visual development and neurocognitive processes such as problem-solving [8, 9]. Arachidonic acid (ARA) is suggested to be essential for growth, brain development, and function as a precursor of bioactive mediators [10]. PUFAs also contribute to sensory and neuronal maturation and play a key role in immune function by modifying cell-membrane composition, signaling pathways, and the production of inflammation-regulating compounds [11, 12].

On average, human milk contains 3.8–3.9 g/100 ml fat, which covers about 50% of the daily energy requirement, with the remaining 50% provided by carbohydrates and proteins [6]. Fatty acids in human milk are originating from both mammary gland production and maternal dietary intake. A substantial proportion of milk SFAs, particularly SCFA and medium-chain fatty acids (MCFA) are synthesized *de novo* in the mammary gland from non-lipid precursors, whereas long-chain fatty acids, including most MUFAs, PUFAs, and trans-fatty acids (TFAs), are transferred from maternal plasma and reflect dietary intake and adipose tissue storage [6]. PUFAs such as linoleic acid (LA) and alpha-linoleic acid (ALA) are fully dependent on dietary intake and body storage, while ARA, eicosapentaenoic acid (EPA) and DHA can be formed out of LA and ALA, but only in small amounts [4, 13, 14]. The main dietary source of DHA and EPA are fish and sea food, as well as poultry and eggs, although they contain DHA at a lower level compared to fish. The most important sources for ALA are soybean and canola oils, as well as flax seed oil and some nuts [15].

Maternal diet thus has an important role in the supply of fatty acids to the infant. Consequently, the association between maternal diet and human milk fatty acid composition has been studied extensively. In line with earlier research [16], a recent review summarized the available evidence and concluded that maternal fish intake was overall positively associated with human milk ALA, DHA and EPA content. PUFAs concentrations in human milk were generally positively correlated with their maternal dietary intake, while saturated fatty acid intake was negatively correlated with PUFAs in human milk [17]. Yet, studies on micronutrient intake and associations with other fatty acids remain inconclusive. Milk lipid production depends on the integration of endogenous fatty acid synthesis, uptake of circulating lipids, and efficient triglyceride assembly and secretion [18, 19]. Micronutrients support several aspects of these processes by maintaining cellular metabolism, enabling enzymatic reactions, regulating gene expression, and protecting lipids from oxidative damage [20, 21]. Consequently, maternal nutritional status may influence both the quantity and composition of milk lipids, which in turn can affect infant nutrition and development. In addition, studies often lack strict sampling protocols. Such milk sampling and processing protocols are essential, as human milk fatty acid content is dynamic and varies with time postpartum, timing within and between feeds, and time of day [22, 23]. These sources of variability complicate accurate measurement and make strict sampling procedures important for increasing estimate precision and decreasing the risk of sampling error.

Thus, information on the role of maternal diet in the supply of fatty acids other than PUFAs is so far missing. Therefore, we aimed to study the association of maternal diet on human milk fatty acid composition, by setting up a study that followed strict sampling protocols.

## Methods

We conducted a cross-sectional study to assess the association between maternal dietary intake and human milk fatty acid composition—the MELK study. The study aimed to recruit 120 healthy lactating women, living in The Netherlands between July 2022 and December 2024. The study protocol was approved by the Medical Ethical Committee Oost-Nederland (NL79447.091.21) and all participants provided written informed consent before entering the study. Sample size calculations were performed using G*Power. The sample size calculation was described previously in the study design paper [24]. Briefly, based on a reported correlation of 0.33 between maternal DHA intake and human milk DHA concentration, a sample size of 109 participants was required to achieve 95% power at a two-sided α of 0.05. Allowing for 10% drop out, the target recruitment was set at 120 lactating women. A detailed study protocol of the full cohort has been described previously [24].

### Study design

Mothers were recruited at 2 months postpartum through midwives, maternity care specialists and social media. Mothers were enrolled if they were ≥ 18 years old, had a pre-pregnancy BMI between 18.5 and 24.9 kg/m², and were exclusively breastfeeding. Infants were included if they were delivered at term (37–42 weeks), had a birth weight ≥ 2.5 kg, were apparently healthy, and had not received antibiotics.

Data collection was conducted over five scheduled study days within a four-week period. During this period, participants provided two milk samples (20 ml of a full expression) and one 24 h urine sample and completed questionnaires and dietary assessments. Anthropometric measurements were obtained for both mothers and infants during home visits, and infant growth data were supplemented by information from the routinely collected growth records. Questionnaires captured maternal demographics, lifestyle factors (including physical activity, stress, and sleep), and infant feeding behaviors, gastrointestinal symptoms, and developmental milestones.

### Human milk sampling

Human milk samples were collected on two separate days in the morning between 6:00 and 8:00, following a standardized cleaning and collection protocol, including washing of pump, breast and hands, to minimize variability. Participants were not fasted, as mothers followed their usual feeding and eating routines, including nocturnal breastfeeding and occasional night-time food intake. Participants were instructed to collect a full expression from one breast, using a provided manual pump, of which 20 ml was transferred into a sterile test tube and stored in the household fridge. At the laboratory, milk was skimmed by centrifuging samples at 1831 x *g* for 15 min at 2 °C and fat and water fractions were stored separately.

### Human milk fatty acids compositional analysis

Fatty acid composition was analysed by Eurofins Food Testing Netherlands BV using the ISO 16958:2015 Milk, milk products, infant formula and adult nutritionals - Determination of fatty acids composition - Capillary gas chromatographic method. Briefly, the fat sample was directly trans-esterified, and the resultant fatty acid methyl esters (FAMEs) were subjected to gas chromatography (GC) for separation and analysis. Fatty acid identification was based on comparing retention times with known standards, and quantification was performed using specific response factors for each fatty acid.

### Dietary assessment

Maternal dietary intake was assessed using four 24-hour food records of non-consecutive days, supported by a short food frequency questionnaire (FFQ) to capture habitual intake of less frequently consumed foods. Participants logged foods and beverages in Traqq^®^, a validated mobile app in real time, including portion sizes, brand names, and preparation methods [25]. Nutrient intake was calculated from these records using the Dutch Food Composition Database (NEVO) 2021, allowing quantification of macronutrient and micronutrient intake. Additionally, urinary biomarkers (24-hour urine samples) were collected to validate self-reported dietary intake for protein and potassium. Total 24-hour urinary nitrogen excretion was quantified using the Kjeldahl method with a Foss Kjeltec 2300 analyzer. Urinary potassium concentrations were measured using an ion-selective electrode on a Roche 917 analyzer (Roche Diagnostics). Total 24-hour potassium excretion was calculated by multiplying the total urine volume collected over 24 h by the measured potassium concentration and dividing by 0.81, based on the assumption that 81% of dietary potassium intake is excreted in urine [26]. Urinary protein excretion was estimated using the formula: 6.25 × (urinary nitrogen/0.81), where the correction factor accounts for non-urinary nitrogen losses through feces and skin, estimated at approximately 19% [26]. Validity of 24-hour dietary recall was assessed against 24-hour urinary biomarkers of protein and potassium. Correlations were evaluated using Spearman’s rank correlation. Agreement between reported intake and urinary estimates was assessed using percentage agreement (recall ÷ biomarker × 100). Habitual intake was defined as the average across the four 24-hour food records. The quality of the maternal dietary intake was measured using the DHD-P index [27]. The DHD-P index is a modified version of the Dutch Healthy Diet 2015 index that accounts for the nutritional needs of pregnant women. Participants received a score between 0 and 10 for each of the 19 elements of the DHD-P index, leading to a maximum score of 190.

### Statistical analysis

Descriptive analyses of the study population were conducted, applying basic summary statistics, such as mean and standard deviation (SD) for normally distributed variables. No missing data were present for the variables included in the present analyses. Consequently, all participants were included in the analytic sample. To account for day-to-day variation in human milk fatty acid content, the average of the fatty acid data of the two milk samples was used for analysis. Fatty acid data was expressed as the fraction of total fatty acids (%FA), and the association between maternal diet and human milk fatty acid composition was assessed using beta regression. Beta regression was chosen because it is specifically designed for modelling continuous outcomes bounded between 0 and 1 (such as proportions), allowing for flexible modelling of heteroscedasticity and skewness in the distribution [28]. Given the high number of statistical tests performed, p-values were corrected for multiple comparisons using the Benjamini-Hochberg false discovery rate (FDR) procedure to control for type I error. Total fat intake was expressed as g/L and the association of maternal diet and total fat intake was analyzed by linear regression. To account for differences in total energy intake, all dietary intake variables were energy adjusted before regression using the energy density approach. Nutrient intakes were expressed per 1000 kcal. Potential confounders or effect modifiers were identified using Directed Acyclic Graphs (DAGs) (appendix 2) [29], including maternal and child characteristics as well as sampling procedure. Models were adjusted for maternal age, pre-pregnancy BMI, and parity, as these are the most commonly reported and biologically plausible confounders in the association between maternal diet and human milk fatty acid composition. This covariate set was selected a priori to avoid an overly complex model and reduce the risk of overadjustment given the number of exposures and outcomes assessed. All statistical analyses were performed with statistical software package R 4.5.2.

## Results

Out of 120 recruited participants, 11 dropped out, several of them due to low milk production. Thus, the final study population included a total of 109 mother-infant pairs. Mothers had a mean age of 32.3 years, a mean BMI of 22.1 kg/m^2^ and all of them were highly educated. Infants had a mean gestational age of 40 weeks, with an average birthweight of 3561 g and birth length of 50.3 cm (Table 1).

**Table 1:** Characteristics of 109 mother-infant pairs participating in the study

Maternal characteristics	Mean ± SD
Age	32.3 ± 2.3
BMI (current)	22.1 ± 1.9
BMI (pre-pregnancy)	22.1 ± 1.7
Parity	1.8 ± 0.8
Paid work	100%
Education - high/scientific	100%
Chronic disease	6.4%
Smoking before pregnancy	2.8%
Smoking currently	12.5%
Alcohol before pregnancy	73.4%
Alcohol currently	14.7%
Supplement use	75.2%

Infant characteristics	Mean ± SD
Gestational age (weeks)	40.0 ± 1,0
Birth weight (g)	3561 ± 399
Birth length (cm)	50.3 ± 5.6
Birth head circumference (cm)	33.6 ± 7.2
Weight at 4 weeks (g)	4500 ± 558
Length at 4 weeks (cm)	55.0 ± 1.9
Breastfeeding time per day (min)	155 ± 89.9

The mean total fat content in human milk was 4.03 g/100mL (Table 2). The concentration of MUFAs in the human milk was on average 43.57% with oleic acid contributing 38.64% to total fatty acids. Omega 3 fatty acids contributed 1.85%, with ALA adding 1.25%, DHA 0.22% and EPA 0.09%. Omega 6 fatty acids constituted 14.23%, with LA and ARA contributing 13.04% and 0.38% respectively, 39.45% of all fatty acids in human milk were saturated fatty acids with 20.39% of palmitic acid (Table 2).

**Table 2:** Macronutrient and fatty acid content of the milk of 109 lactating women participating in the study, average of two milk samples

Nutrient	Mean ± SD
Total fat (g/100mL)	4.03 ± 1.05
Total carbohydrate (g/100mL)	7.70 ± 0.25
Total protein (g/100mL)	0.98 ± 0.14
Total SFAs (%FA)	39.45 ± 5.38
Total MUFAs (%FA)	43.57 ± 3.50
Total TFAs (%FA)	0.82 ± 0.29
Total PUFAs (%FA)	16.35 ± 3.92
Total n-6 PUFAs (%FA)	14.23 ± 3.59
Total n-3 PUFAs (%FA)	1.85 ± 0.65
SFAs	
Butyric acid (C4:0) (%FA)	0.13 ± 0.16
Caproic acid (C6:0) (%FA)	0.09 ± 0.03
Caprylic acid (C8:0) (%FA)	0.15 ± 0.05
Capric acid (C10:0) (%FA)	1.22 ± 0.28
Lauric acid (C12:0) (%FA)	4.68 ± 1.70
Myristic acid (C14:0) (%FA)	5.26 ± 1.44
Palmitic acid (C16:0) (%FA)	20.39 ± 3.21
Stearic acid (C18:0) (%FA)	6.54 ± 1.69
Arachidic acid (C20:0) (%FA)	0.19 ± 0.05
Behenic acid (C22:0) (%FA)	0.07 ± 0.03
Lignoceric acid (C24:0) (%FA)	0.09 ± 0.08
MUFAs-cis	
Palmitoleic acid (C16:1 n-7) (%FA)	1.91 ± 0.53
Vaccenic acid (C18:1 n-7) (%FA)	1.56 ± 0.24
Oleic acid (C18:1 n-9) (%FA)	38.64 ± 3.50
Caproleic acid (C10:1) (%FA)	0.00 ± 0.00
MUFAs-trans	
Palmitelaidic acid (C16:1 n-7) (%FA)	0.10 ± 0.05
Trans-vaccenic acid (C18:1 n-7) (%FA)	0.25 ± 0.13
Elaidicnic acid (C18:1 n-9) (%FA)	0.08 ± 0.03
PUFAs	
γ-Linolenic acid (C18:3 n-6) (%FA)	0.11 ± 0.03
Arachidonic acid (C20:4 n-6) (%FA)	0.38 ± 0.11
Linoleic acid (C18:2 n-6) (%FA)	13.04 ± 3.52
α-Linolenic acid (C18:3 n-3) (%FA)	1.25 ± 0.45
Eicosapentaenoic acid (C20:5 n-3) (%FA)	0.09 ± 0.09
Docosahexaenoic acid (C22:6 n-3) (%FA)	0.22 ± 0.19

The average daily energy intake of the lactating women was 2277 kcal, with an average daily fat intake of 97.0 g (Table 3). The mean intakes of SFAs, MUFAs, and PUFAs were 13.18 en%, 14.18 en%, and 7.66 en%, respectively. Among PUFAs, n-6 fatty acid intake averaged 6.58 en%, while n-3 fatty acid intake was 0.90 en%. Total DHD-P score was low, with 93.5 out of 190. The lowest scoring categories were unhealthy products, vitamin D, fish and salt intake (data not shown). Monthly fish intake as assessed by FFQ was also low (1.42 g/day). The Spearman correlation between mean of four records and urinary excretion of potassium was ρ = 0.48, *p* < 0.001, with a mean percentage agreement of 121%, indicating some overestimation by recall. For protein, calculated from nitrogen excretion, the correlation was ρ = 0.42, *p* < 0.001, with a mean percentage agreement of 115%, also suggesting modest over-reporting.

**Table 3:** Average maternal dietary intake data of 109 women, collected through four 24 h food records

Dietary intake	Mean ± SD
Total energy (kcal/day)	2277 ± 427
Total protein (en%/day)	15.0 ± 2.6
Total carbohydrates (en%/day)	44.9 ± 5.7
Total dietary fibre (en%/day)	2.4 ± 0.6
Total fat (en%/day)	37.2 ± 5.1
Saturated fatty acids (en%/day)	13.18 ± 2.80
Monounsaturated fatty acids cis (en%/day)	14.2 ± 2.4
Trans fatty acids (en%/day)	0.3 ± 0.1
Polyunsaturated fatty acids (en%/day)	7.7 ± 2.3
n-6 PUFA (en%/day)	6.6 ± 2.0
n-3 PUFA (en%/day)	0.9 ± 0.4
EPA from fish (FFQ) (g/day)	0.05 ± 0.05
DHA from fish (FFQ) (g/day)	0.09 ± 0.08
Sodium (mg/day)	2365 ± 992
Potassium (mg/day)	3326 ± 1,031
Magnesium (mg/day)	410.3 ± 133.3
Zinc (mg/day)	10.7 ± 2.5
Copper (mg/day)	1.9 ± 0.6
Retinol (µg/day)	497.1 ± 938.4
Vitamin B1 (mg/day)	1.1 ± 0.3
Vitamin B2 (mg/day)	1.4 ± 0.4
Vitamin B6 total (mg/day)	1.4 ± 0.4
Vitamin B12 (µg/day)	3.7 ± 2.0
Vitamin E total (mg/day)	15.0 ± 4.6
Vitamin C (mg/day)	96.9 ± 42.3
Niacin (mg/day)	14.7 ± 4.5
Vitamin D total (µg/day)	2.6 ± 1.5
Folate (µg/day)	301.0 ± 87.0
Vitamin K total (µg/day)	178.1 ± 161.2
Total DHD-P score (max 190)	93.54 ± 15.07
Total fish intake (g/day)	1.42 ± 3.05

**Fig. 1:**
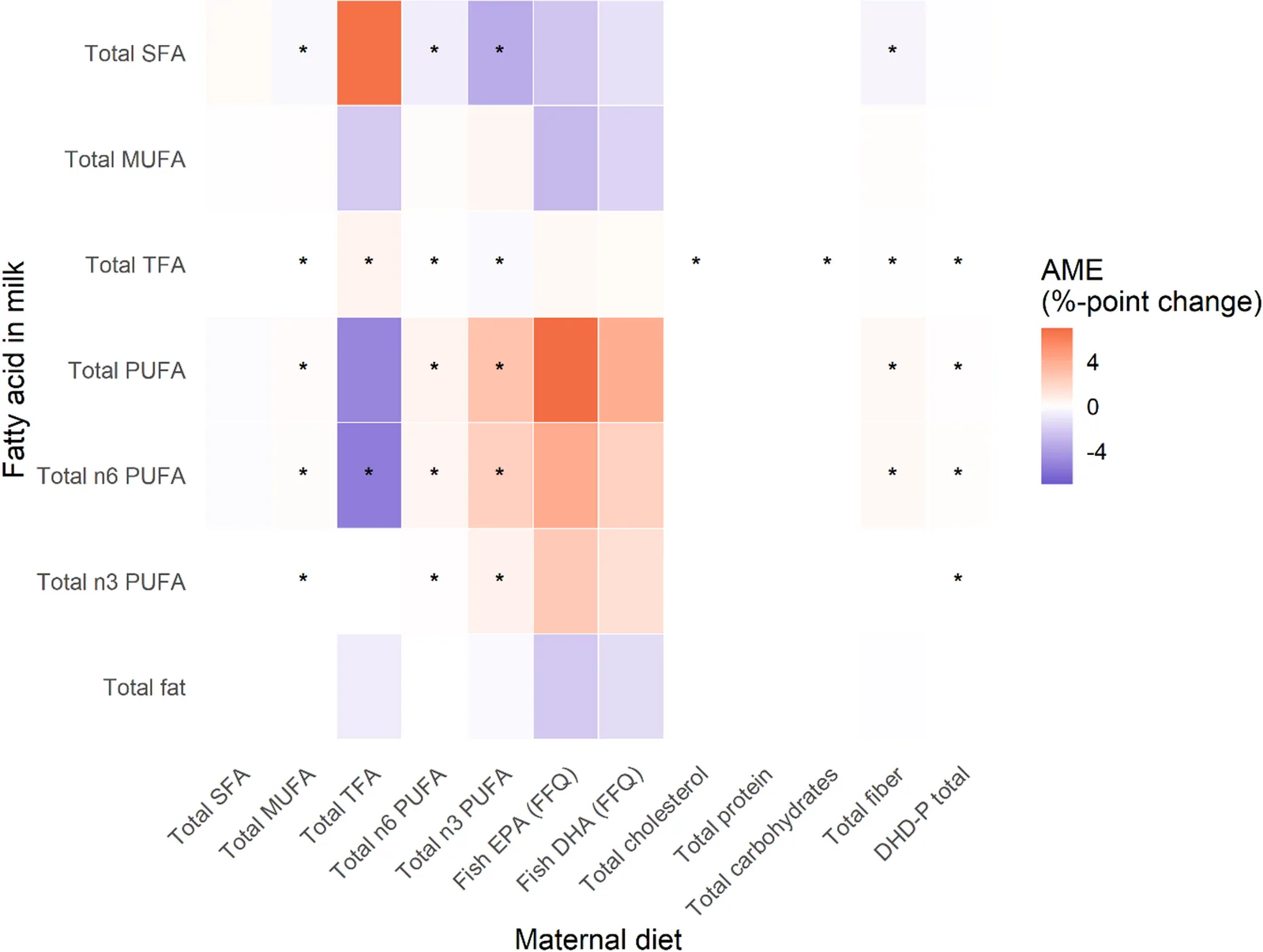
Results of beta regression analysis—changes in milk fatty acid groups according to macronutrient intake. AME: Average Marginal Effect. Red shading: positive associations; blue shading: negative associations. size of the associations, expressed as %-point change in milk fatty acids per 1-unit increase in macronutrient intake (per 1000 kcal). *: FDR adjusted statistical significance. Adjusted for maternal age, pre-pregnancy BMI and parity

Maternal macronutrient intake was associated with several fatty acid groups in human milk (Fig. 1, for full models see appendix 1). A 1 g/day higher intake of n-3 PUFAs was associated with a higher proportion of n-3 fatty acids in human milk (0.63%-points, CI (0.45– 0.80)), and this did not change when n-3 supplementation was taking into account (0.57%-points, CI (0.44–0.70)). Higher dietary n-3 PUFAs was also associated with a higher proportion of total PUFAs and n-6 PUFAs (2.29%-points, CI (1.14–3.43)) and lower proportions of SFAs and TFAs (− 3.43%-points, CI (− 5.33 to − 1.53); − 0.21%-points, CI (− 0.34 to − 0.09), respectively). In line with that, higher n-6 PUFA intake was associated with slightly higher proportions of milk n-3 PUFAs (0.08%-points, CI (0.05–0.11)), as well as n-6 PUFA (0.49%-points, CI (0.33–0.66)), and total PUFAs. As for n-3 PUFAs, negative associations of dietary n-6 PUFAs with SFAs and TFAs were seen.

**Fig. 2:**
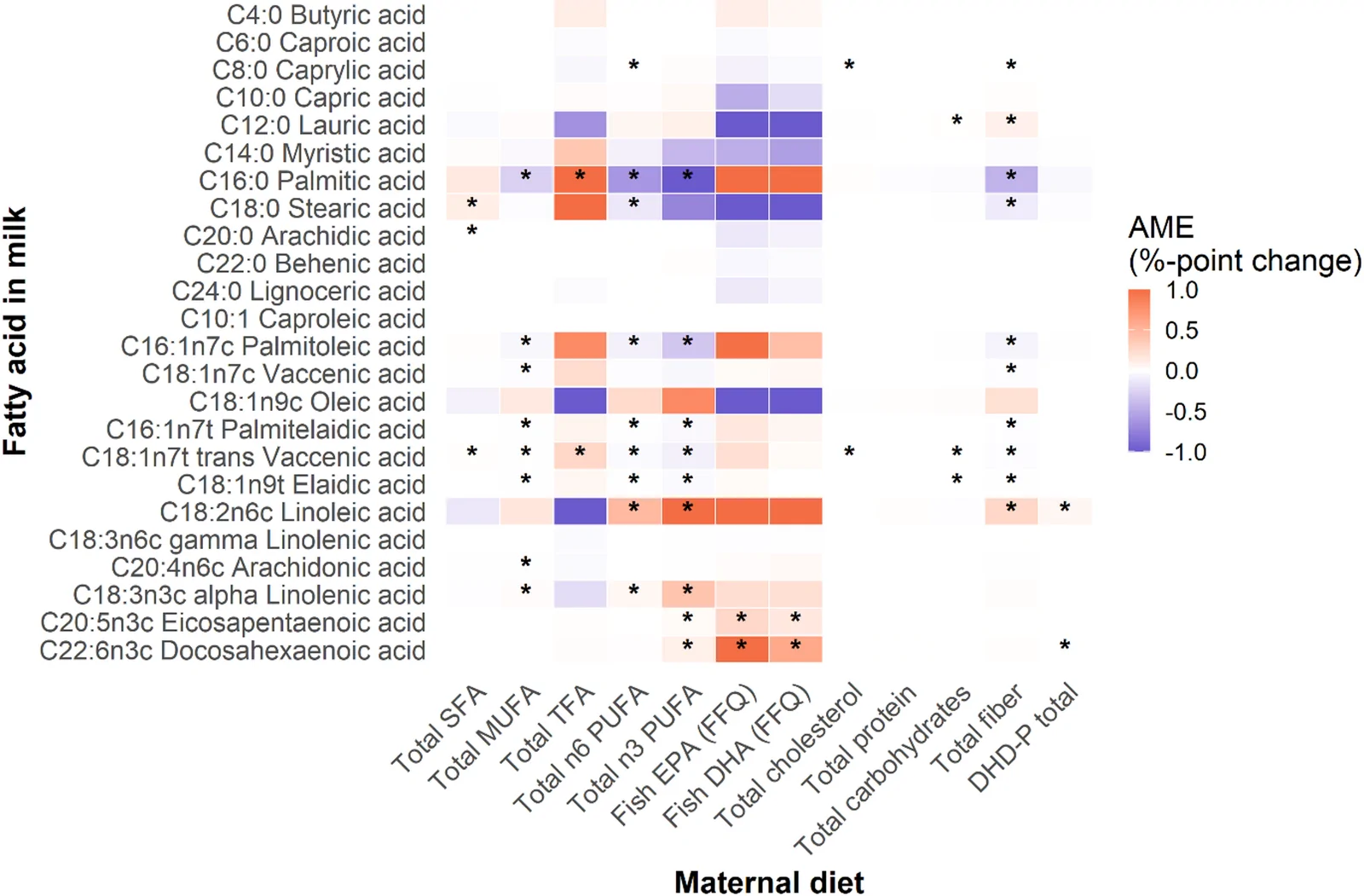
Results of beta regression analysis—change in milk fatty acids according to macronutrient intake. AME: Average Marginal Effect. Red shading: positive associations; blue shading: negative associations. size of the associations, expressed as %-point change in milk fatty acids per 1-unit increase in macronutrient intake (per 1000 kcal). *: FDR adjusted statistical significance. Adjusted for maternal age, pre-pregnancy BMI and parity

Maternal n-3 PUFAs intake demonstrated the clearest associations with individual fatty acids, too (Fig. 2, table S1/S2). Maternal n-3 PUFAs intake was positively associated with milk n-3 fatty acids, including ALA (0.2%-points, CI (0.29–0.54)), DHA (0.1%-points, CI (0.04–0.17)) and EPA (0.4%-points, CI (0.01–0.06)). Its intake was also positively associated with the n-6 PUFA LA. Maternal n-6 PUFAs intake displayed a similar profile, with positive associations for LA (049%-points, CI (0.32–0.65)) and ALA (006%-points, CI (0.04–0.08)) proportions in milk, but negative associations with palmitic acid (− 062%-points, CI (− 0.78 to − 0.46)) and stearic acid (− 013%-points, CI (− 0.23 to − 0.04)). Higher intake of n-3 and n-6 PUFA also showed inverse associations with the proportions of several TFA. DHA and EPA intake measured by FFQ did not show an association with proportions of fatty acid groups in milk, but were positively associated with DHA and EPA proportions (Figs. 1 and 2).

While TFA intake was associated with total TFAs proportions in milk (Fig. 1), intake of total SFAs was not associated with its total level in milk. Yet, on individual fatty acid level, higher maternal SFA intake was positively associated with the SFA stearic acid (0.12%-points, CI (0.05–0.19)) and arachidic acid (0.003%-points, CI (0.001–0.01)) as well as trans-vaccenic acid (0.0%-points, CI (0.003–0.02)) (Fig. 2).

Besides fat intake also other macro-nutrients were observed to be associated with human milk fatty acid composition. Intake of dietary fiber was positively associated with the proportion of total human milk PUFAs and n-6 PUFAs and inversely with the fractions of SFAs and TFAs (Fig. 1). This was partly reflected by associations with individual fatty acids. Fiber intake was positively associated with LA and several MCFA, such as lauric and caprylic acid, and inversely associated with palmitic and stearic acid the MUFAs palmitoleic acid as well as several TFAs (Fig. 2).

Total carbohydrate intake showed mixed associations, including a positive association with lauric acid, and a negative association with trans vaccenic acid and elaidic acid.

Diet quality was weakly associated with milk fatty acid groups. A higher DHD-P score, thus an overall healthier diet, was associated with higher proportions of n-3 (0.01%-points, CI (0.004–0.02), n-6 (0.07%-points, CI (0.02–0.11)) and total PUFAs (0.08%-points, CI (0.03–0.12)) and lower TFAs (− 0.01%-points, CI (− 0.01 to − 0.001)). Adherence to the DHD-P was also weakly positively associated with proportions of DHA (0.004%-points, CI (0.002–0.01)) and LA (0.06%-points, CI (0.02–0.10)) (Fig. 2). For full models see appendix 1.

**Fig. 3:**
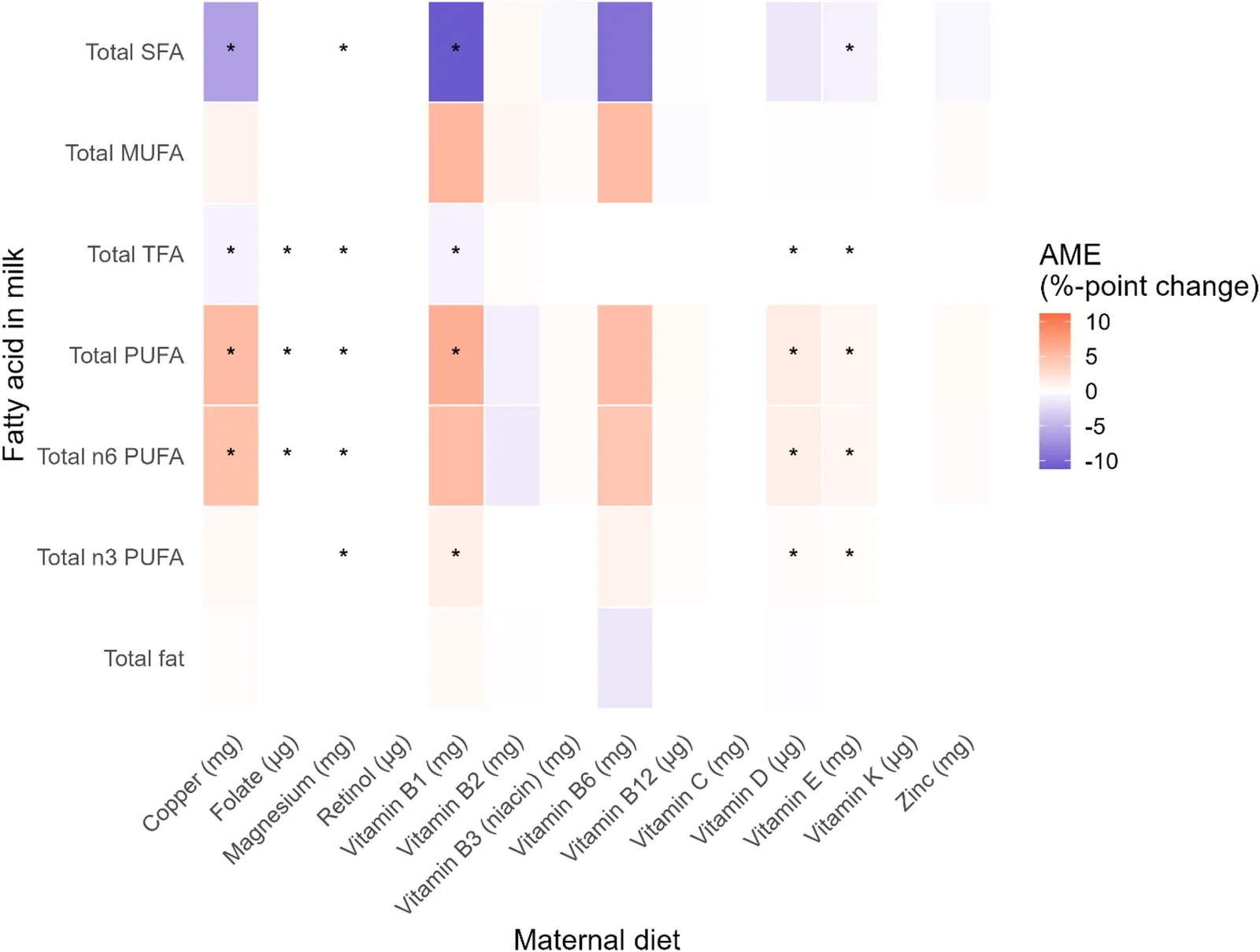
Results of beta regression analysis—change in milk fatty acid groups according to micronutrient intake. AME: Average Marginal Effect. Red shading: positive associations; blue shading: negative associations. size of the associations, expressed as %-point change in milk fatty acids per 1-unit increase in macronutrient intake (per 1000 kcal). *: FDR adjusted statistical significance. Adjusted for maternal age, pre-pregnancy BMI and parity

**Fig. 4:**
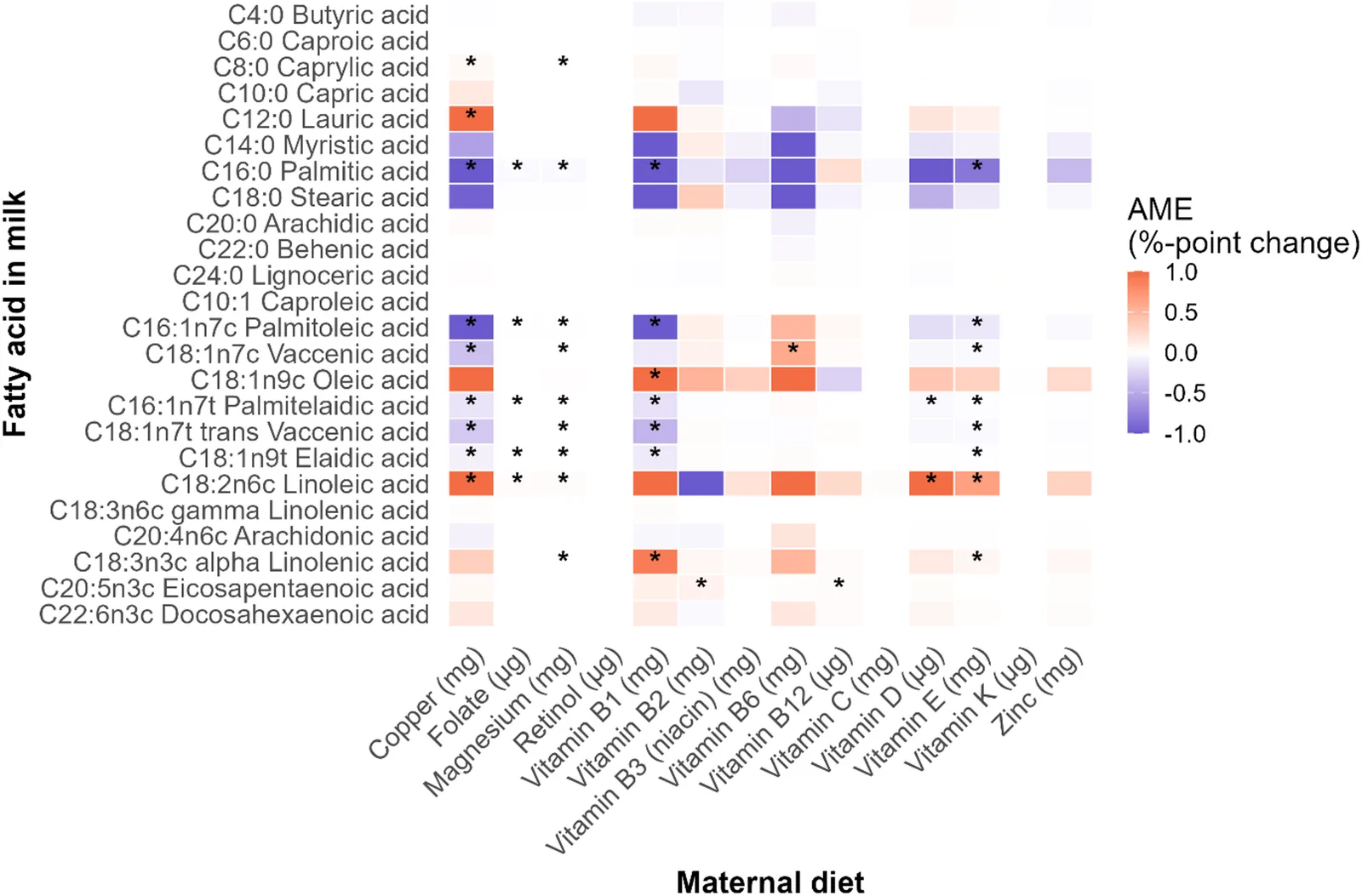
Results of beta regression analysis—change in milk fatty acids according to micronutrient intake. AME: Average Marginal Effect. Red shading: positive associations; blue shading: negative associations. size of the associations, expressed as %-point change in milk fatty acids per 1-unit increase in macronutrient intake (per 1000 kcal). *: FDR adjusted statistical significance. Adjusted for maternal age, pre-pregnancy BMI and parity

In addition to dietary macronutrients, we also investigated associations between intake of micronutrients and milk fatty acids (Fig. 3, for full models see appendix 1). Higher intakes of vitamin B1, copper, vitamin E, and magnesium were each associated with lower proportions of SFAs and TFAs. Folate and vitamin D also showed inverse associations with TFAs proportions. Results regarding the individual fatty acids in milk were in line with this: higher maternal intake of vitamin B1, copper, vitamin E, folate, and magnesium were associated with lower proportions of palmitic acid, palmitoleic acid, and elaidic acid, as well as reduced proportions of palmitelaidic (Fig. 4).

In contrast to the inverse associations observed for SFAs and TFAs, several micronutrients showed positive associations with PUFAs fractions. Higher intakes of magnesium, vitamin E, vitamin D, and vitamin B1 were each positively associated with the proportion of n-3 PUFAs. For n-6 PUFAs, positive associations were observed for magnesium, folate vitamin E, vitamin D, and copper (Fig. 3). Specifically, ALA proportions increased with higher magnesium, vitamin E and vitamin B1 intake (Fig. 4). LA proportions increased with higher magnesium, folate, vitamin E, vitamin D and copper intake. Milk EPA proportion was positively associated with intakes of vitamin B12 and of vitamin B2. Results for MCFAs varied, caprylic acid was positively associated with magnesium and copper intake, and proportions of lauric acid increased only with copper intake. Oleic acid was positively associated with vitamin B1 intake, but not with other intakes of micronutrients (Fig. 4). For full models see online appendix 1.

## Discussion

In this study, both macronutrient and micronutrient dietary intakes in lactating women were examined in relation to the fatty acid profile of their human milk. Dietary n-3 PUFAs, n-6 PUFAs, and TFAs were positively associated with their respective proportions in human milk, and total milk PUFAs was higher with higher intakes of both PUFA classes. Although milk SFAs were not associated with dietary SFAs, it was inversely associated with the intake of most other fatty acid groups, whereas MUFAs proportions in milk were general not associated with diet. Higher diet quality and fiber intake were also linked to higher proportions of milk PUFAs. At the level of individual fatty acids in milk, SFA intake was positively associated with some milk SFAs, and TFAs intake showed a positive association with palmitic acid, while n-3 PUFAs intake showing the opposite pattern. Micronutrient intakes, notable vitamin B1, vitamin D and copper, were generally positively associated with milk PUFAs and negatively associated with SFAs and TFAs in milk.

Several studies have reported dietary n-3 PUFAs, n-6 PUFAs, total PUFAs, SFAs and TFAs to be positively associated to their respective proportions in human milk [17]. Our study confirms these findings, although only individual SFAs and not total SFAs was associated with its dietary intake. As expected, the essential fatty acids LA and ALA in milk were positively associated with the mothers’ intake of n-3 PUFAs. Also EPA and DHA in milk showed clear links with dietary n-3 PUFAs intake. Only ARA in milk was not clearly associated with n-3 intake. These results are partially in line with a French study on colostrum, which reported positive associations with n-3 PUFAs intake and EPA and DHA in milk. In the same study the ratio of ARA to DHA was negatively affected by n-3 intake [30]. Similarly, among South Korean mothers, Kim et al. found that milk EPA and DHA were associated with n-3 intake [31]. In our study, intake of carbohydrates was only associated with TFA in milk, and protein did not show any association with fatty acids in human milk. In the study of Kim et al., carbohydrate intake was also associated with SFAs, MUFAs and the n-6 to n-3 PUFA ratio [31], and another study reported correlations of carbohydrate intake with total PUFAs, n-6 PUFAs, MUFAs, LA and ARA in milk [32]. Similarly, some studies reported correlations between protein intake and several milk PUFAs [33, 34], but most studies, including ours, did not find any [17].

Besides dietary macronutrients we also investigated dietary micronutrients and observed associations with several fatty acids in milk. Proportions of milk SFAs and TFAs were generally lower with higher micronutrient intakes, while proportions of total PUFAs, as well as of n-3 and n-6 PUFAs in milk, were positively associated. Studies addressing the role of micronutrients regarding fatty acids in human milk are scarce. The observational study of Butts and colleagues [32] assessed a variety of micronutrients, pointing out positive correlations between folate intake and DHA, LA and n-3 PUFAs in milk. Our results are partly in line with that, as LA in milk was weakly positively associated with folate intake in our study, but neither DHA nor n-3 PUFA were associated. Yet, due to a lack of a measure of significance in the paper by Butts et al. the results are difficult to compare. Also, unlike our study, they did not observe associations of magnesium, vitamin B1 and vitamin D intake with milk fatty acids [32]. Copper, vitamin B6 and vitamin E were studied by us, but not by Butts et al. Thus, comparable data on the role of micronutrients from maternal diet on fatty acids is milk are still limited.

Fatty acids in milk triglycerides originate from three primary sources: dietary lipids, maternal adipose tissue stores, and fatty acids formed *de novo* from glucose and other precursors in the mammary gland [19]. De novo lipogenesis in the mammary gland is responsible for the synthesis of particularly SCFAs and MCFAs [35]. In addition to *de novo* synthesis, circulating triglycerides, containing longer chain fatty acids, such as PUFAs derived from maternal diet or adipose tissue, can be converted into free fatty acids after which they can be taken up by mammary epithelial cells for incorporation into milk lipids [18]. Thus, essential fatty acids are directly stemming from maternal diet or from fat reserves, explaining the observed associations found between n-3, n-6 and total PUFAs intake and these fatty acids in milk.

Because milk lipid synthesis requires high levels of energy, substrate supply, and enzymatic activity, micronutrient availability may influence the efficiency of these metabolic processes [18, 19]. In a randomized controlled trial of 70 mothers, supplementation with multiple micronutrients alongside DHA has been shown to increase the DHA content of human milk compared to placebo. The supplement in this trial consisted of 200 mg DHA, alongside 3600 IU vitamin A, 60 mg vitamin C, 600 IU vitamin D, 10 IU vitamin E, 1.4 mg vitamin B1, 1.6 mg vitamin B2, 17 mg vitamin B3, 7 mg vitamin B5, 2 mg vitamin B6, 2.8 µg vitamin B12, 500 µg folic acid, 35 µg biotin, 120 mg calcium, 225 µg iodine, 9 mg iron, 55 µg selenium, 10 mg zinc, and 250 µg lutein [36]. This study suggests that micronutrient intake could alter the fatty acid profile of milk in lactating women. However, the exact contribution of micronutrient intake was not separated from the influence of the DHA supplements [36]. Vitamin E (α-tocopherol) is present both in diet and in human milk. In a study of milk from lactating mothers, α-tocopherol in milk was positively associated with measures of antioxidant activity in human milk, suggesting that vitamin E helps protect milk lipids, including PUFAs, from oxidative damage [21]. Other micronutrients, including B-vitamins, copper, and magnesium, are essential cofactors in many metabolic and enzymatic processes involved in cellular lipid metabolism. Deficiencies in these nutrients have been associated with alterations in lipid metabolism in experimental and clinical studies, but evidence for direct effects of variations in intake of these micronutrients on the fatty acid composition of human milk remains limited [20, 37, 38]. Thus, milk fat composition arises from coordinated lipid synthesis, uptake, and secretion in the mammary gland, processes that depend on sufficient micronutrient support. Maternal nutritional status can therefore influence both the amount and quality of milk lipids, with implications for infant development. Yet, whether micronutrient intake from diet is a truly limiting factor in the synthesis of fatty acids or if micronutrient intake rather reflects an overall health status of a mother remains unclear. Foods rich in copper include seafood, liver, cocoa products, nuts and seeds, while vitamin B1 is especially abundant in whole grains, pulses, meat, liver and fish [39, 40]. Vitamin E is highest in vegetable oils, fat spreads from vegetable oils, nuts and seeds, some fatty fish, egg yolk and whole grain cereals [41].

When studying human milk composition, especially fatty acids, it is important to also consider factors other than diet that may affect the fatty acid profile. Maternal body composition is considered an important factor, as the mothers’ adipose tissue stores can release fatty acids for incorporation in milk. A systematic review found that besides leptin and insulin, the ratio of n-3 to n-6 PUFAs of human milk was affected by maternal BMI but results for other fatty acids varied [42]. Another physiological factor is the activity of the enzymes FADS1 and FADS2, with FADS1 polymorphism linked to the content of ARA, EPA and DHA in milk [43–45]. Besides these physiological attributes, human milk fatty acid content is further influenced by the time postpartum, with higher fat content in colostrum and a decrease of long chain PUFAs over time [46]. Yet other factors influencing human milk compositions may lie in behavioral characteristics such as stress [47] and smoking [48], or ethnic differences between women [49].

In this study we used repeated standardized dietary assessments, which reduces measurement error and captures habitual intake as well as within-mother variation. We also followed a strict sampling protocol, that ensured high comparability between participants by pre-defining the time postpartum, excluding mothers with a health condition and applying the same breast pump. Lastly, we controlled for several key external influences (maternal age, pre-pregnancy BMI and parity) and collected additional data to examine specific factors influencing the associations of interest, such as the time of the day and volume of milk expressed. While our study followed strict sampling and analytical protocols, some limitations should be considered when interpreting the findings. Although the sample size was based on a priori calculations using the established association between maternal DHA intake and milk DHA content, a larger sample may be required to detect weaker associations for other dietary components that might be part of more complex biological pathways. While participants were recruited non-selectively, the final study sample consisted largely of highly educated women with generally healthy lifestyles, which limits the generalizability of the results to the broader Dutch population. This relative homogeneity also makes it difficult to disentangle the effects of individual nutrients from the overall pattern of a healthy diet. For example, dietary fiber intake was positively associated with milk PUFAs, particularly n-6 PUFAs, but this association may reflect fiber as a marker of a healthier diet, based on whole grains and vegetables, rather than a direct biological effect on milk PUFAs composition. An additional limitation is that milk samples were collected at a single time point rather than over a 24 h period. Human milk fat content is known to vary throughout the day [50]. Consequently, our sampling approach does not reflect daily variation in total milk lipid content. This study focused on the milk fatty acid profile, with fatty acids expressed as a percentage of total fatty acids. Accordingly, the findings reflect the relative composition of fatty acids in the collected milk samples rather than absolute lipid or fatty acid concentrations. Participants were asked to collect a full milk expression and submit a 20 ml aliquot after mixing the expressed sample. However, differences in sample handling and homogenization prior to aliquoting may have contributed to between-sample variability. Additionally, maternal blood fatty acid biomarkers were not available, as blood samples were not collected within the cohort protocol. Such biomarkers could have provided complementary information on maternal fatty acid status and strengthened the interpretation of the relationship between dietary intake and milk fatty acid composition. A further consideration is the use of the DHD-P index to assess maternal diet quality. Although the DHD-P was developed to evaluate adherence to Dutch dietary guidelines during pregnancy, it was applied in the cohort of lactating women. While many dietary recommendations overlap between pregnancy and lactation, lactating women have specific macro- and micronutrient requirements that are not fully captured by the DHD-P. Therefore, the diet quality scores reported in this study should be interpreted as reflecting adherence to broadly healthy dietary recommendations rather than compliance with all nutritional recommendations specific to lactation. Future studies may benefit from the development or application of diet quality indices specifically tailored to lactating populations. Finally, we used biomarkers to examine the validity of our dietary measurement, which showed some over-reporting. This may have inflated absolute nutrient intake values, but patterns of nutrient intake across participants likely remain valid for assessing associations with human milk fatty acids, as correlations of intake with biomarkers were similar to those reported in earlier studies [51, 52].

Our study confirms previous reported associations between fatty acid intake from maternal diet and fatty acids, especially PUFAs in human milk. It also indicates possible roles of dietary micronutrients and vitamins in determining the fatty acid composition of human milk. While micronutrients are involved in several metabolic processes related to fatty acid synthesis, storage and release, it is still unclear if there is a direct relation between maternal micronutrient intake and fatty acid levels in milk, or whether micronutrient intake instead serves as a proxy for a larger broader dietary patterns that influence fatty acid composition.

## Supplementary Information

Below is the link to the electronic supplementary material.

Supplementary Material 1

Supplementary Material 2

## Data Availability

The data are not publicly available due to ethical restrictions related to participant confidentiality. De-identified data may be made available from the corresponding author upon reasonable request, subject to institutional approval.

## Abbreviations

ALA Alpha: linolenic acid
AME: Average marginal effect
ARA: Arachidonic acid
DHA: Docosahexaenoic acid
EPA: Eicosapentaenoic acid
FAME: Fatty acid methyl esters
FDR: False discovery rate
FFQ: Food frequency questionnaire
GC: Gas chromatography
LA: Linoleic acid
MCFA: Medium-chain fatty acids
MUFA: Monounsaturated fatty acids
PUFA: Polyunsaturated fatty acids
SCFA: Short-chain fatty acids
SFA: Saturated fatty acids
TFA: Trans fatty acids
